# Safety and efficacy of oral desensitization to swordfish in children with persistent and multiple fish allergy

**DOI:** 10.1186/2045-7022-3-S3-P21

**Published:** 2013-07-25

**Authors:** G Sampaio, S Piedade, M Morais-Almeida

**Affiliations:** 1 Immunoallergy Department, CUF-Descobertas Hospital, Lisboa, Portugal

## Background

In Portugal, fish allergy is the third cause of food allergy in children and it can be severe and persistent. Patients are often allergic to several fish species, based on the cross-reactivity of their parvalbumins. However, some studies indicate that there are fish species, like swordfish, with lower levels of parvalbumins and which are expected to be of lower allergenicity. Management for this disease is based on allergen avoidance and symptomatic treatment of accidental allergic reactions and there are few studies about oral fish desensitization.

## Methods

In order to document and share experiences we present a protocol of specific oral desensitizing treatment (SODT) to swordfish applied to 2 children, a girl and a boy, with 9 and 10 years old respectively, with persistent IgE-mediated allergy to several fish species. Both had personal history of allergic rhinitis and asthma. The girl had also egg allergy. Swordfish was the first selected fish for oral desensitization. The presence of swordfish allergy was confirmed by a positive oral food challenge. The protocol, using cooked swordfish as allergen extract, began with chew / spit out doses followed by oral ingestion of increasing doses, always in hospital settings, until reaching the target dose of 50g/day. Informed consent was obtained at the beginning and at all treatment sessions, and the telephone number of the medical staff was offered.

## Results

Both children had a positive oral food challenge with immediate urticaria and/or oropharyngeal itching after swordfish oral mucosa contact. The desensitization protocol began twenty minutes later, after spontaneous resolution of the symptoms, and both children achieved 1g/day on the first session. They achieved the target dose of 50g/day in three-four more hospital sessions. During the SODT the two children had mild adverse reactions, occurring in the two first sessions, and with spontaneous resolution.

## Conclusion

Specific and sequential oral desensitization to different fish species may represent an alternative and innovative approach in children with multiple fish allergy.

## Disclosure of interest

None declared.

